# Development of anisotropic phantoms using wood and fiber materials for diffusion tensor imaging and diffusion kurtosis imaging

**DOI:** 10.1007/s10334-019-00761-3

**Published:** 2019-05-29

**Authors:** Masashi Suzuki, Susumu Moriya, Junichi Hata, Atsushi Tachibana, Atsushi Senoo, Mamoru Niitsu

**Affiliations:** 1 Department of Radiological Sciences, Graduate School of Human Health Sciences, Tokyo Metropolitan University, 7-2-10 Higashi-Ogu, Arakawa-ku, Tokyo, 116-8551 Japan; 2 Department of Radiology, Saitama Medical University Hospital, 38 Morohongo, Moroyama, Iruma, Saitama 350-0495 Japan; 3 Sawai Memorial Breast Clinic, 98 Kamikamo-Matsumoto-cho, Kitaku-ku, Kyoto-shi, Kyoto 603-8052 Japan; 4 Laboratory for Marmoset Neural Architecture, Center for Brain Science RIKEN, 2-1 Hirosawa, Wako, Saitama 351-0198 Japan; 5 Applied MRI Research, Department of Molecular Imaging and Theranostics, National Institute of Radiological Sciences, National Institutes for Quantum and Radiological Science and Technology, 4-9-1 Anagawa, Inage-ku, Chiba, 263-8555 Japan

**Keywords:** Anisotropy, Diffusion magnetic resonance imaging/methods, Phantoms imaging, Diffusion tensor imaging/instrumentation

## Abstract

**Objective:**

Several studies have demonstrated that anisotropic phantoms can be utilized for diffusion magnetic resonance imaging. The purpose of our study was to examine whether wood is suitable as an anisotropic phantom material from the viewpoints of affordability and availability. In the current study, wood was used for restricted diffusion, and fibers were used for hindered diffusion.

**Materials and methods:**

Wood and fiber phantoms were made. Diffusion kurtosis images were acquired with three magnetic resonance scanners. Fractional anisotropy, radial diffusivity, axial diffusivity, radial kurtosis and axial kurtosis values were measured. The wood phantom was imaged, and its durability was confirmed. The phantoms were imaged in varying orientations within the magnetic field. The wood was observed using an optical microscope.

**Results:**

Ten kinds of wood and the fiber had a diffusion metrics. The wood diffusion metrics suggested low variation over a period of 9 months. Changing the orientation of the phantoms within the magnetic field resulted in changes in diffusion metrics. Observation of wood vessels and fibers was conducted.

**Discussion:**

Wood and fibers have anisotropy and are promising as phantom materials. The development of anisotropic phantoms that anyone can use is useful for diffusion magnetic resonance imaging research and clinical applications.

## Introduction

In clinical magnetic resonance imaging (MRI) examinations, diffusion tensor imaging (DTI) and diffusion kurtosis imaging (DKI) provide image contrasts that are different from conventional imaging methods, and are useful for diagnosing diseases that are currently difficult to differentiate [1]. MRI creates images using protons, and the *T*1-relaxation and *T*2-relaxation times of human tissue. A diffusion-weighted imaging (DWI) method for proton diffusion motion imaging has been developed [2] and is indispensable for the early diagnosis of stroke [3, 4]. DTI [5], which involves estimation by multivariate regression of water diffusivity with anisotropy, and DKI [6], which can provide a contrast reflecting complicated diffusion motion in vivo, were both developed from DWI.

For clinical applications, there have been studies on DTI and DKI [7, 8], and studies on anisotropic phantoms [9, 10]. However, anisotropic phantoms for DTI and DKI have room for improvement, in particular in relation to cost. For example, a glass capillary J5022-16 (capillary plate was 33 mm in diameter and 30 mm in length, with a hole diameter of 10 µm, Hamamatsu Photonics K.K., Japan) is expensive, at approximately $10,000 or more. Glass capillaries are often used as phantom materials for restricted diffusion [11–14]. In a previous study, capillaries with large capillary diameters had low fractional anisotropy (FA) values and were not able to provide sufficient anisotropy [15].

The aim of the current study was to assess whether wood is suitable as an anisotropic phantom material. The authors focused on wood as a phantom material because it is typically affordable and easy to obtain. The wall of cells constituting the wood vessels and fibers may restrict water molecule diffusion. In the present study, fibers involving hindered diffusion were also evaluated as a phantom material in a similar manner. Novel anisotropic phantoms may advance the clinical application of DTI and DKI, and enable experiments.

## Materials and methods

### Phantoms

Three types of phantoms were made: an FA-diffusivity value phantom, which consisted of five kinds of wood; a radial kurtosis (RK)–axial kurtosis (AK) value phantom, which consisted of an additional five kinds of wood not used in the FA-diffusivity value phantom; and a fiber phantom, which consisted of Tsunooga.

Dyneema (Ultra-High Molecular Weight Polyethylene Fiber, DSM, Geleen, The Netherlands) has been reported as a phantom material able to hinder diffusion [16]. However, in the current study, the authors utilized Tsunooga (High strength polyethylene fiber, Tsunooga is a registered trademark of TOYOBO, TOYOBO, Japan) instead of Dyneema. Tsunooga was chosen to confirm its use as a novel fiber material.

#### Wood phantoms

Available and affordable wood species were chosen and provided by the Forest Research and Management Organization mentioned in the acknowledgement. The exact age of the tree used as the source of the wood was unknown; however, the wood was collected from a sufficiently grown tree. In a preliminary study, we measured the water content inside wood [post-boiling wood weight (g)—pre-boiling dry wood weight (g) = water content inside wood] for 10 types of wood. Detailed information about these wood samples can be found by searching the TWTwNo at [http://db.ffpri.affrc.go.jp/WoodDB/TWTwDB-E/home.php]. Five wood species were used for the FA-diffusivity value phantom: (Wood species, water content (g), TWTwNo); *Gleditsia triacanthos*, 10.9, 21782; *Cinnamomum sieboldii*, 12.8, 14926; *Euodia meliifolia*, 15.0, 12897; *Ilex pedunculosa*, 11.0, 24497; and *Acer palmatum*, 12.8, 19878. Wood from the *Trachycarpus*, 25.8, 2753; *Betula platyphylla*, 11.6, 25057; *Fraxinus longicuspis*, 10.6, 25702; *Eucalyptus*, 13.3, 15615; and *Acer mono* 10.9, 13921 species were used for the RK–AK value phantom. As FA-diffusivity and RK–AK are different, they were analyzed individually and produced as two separate phantoms.

**Fig. 1:**
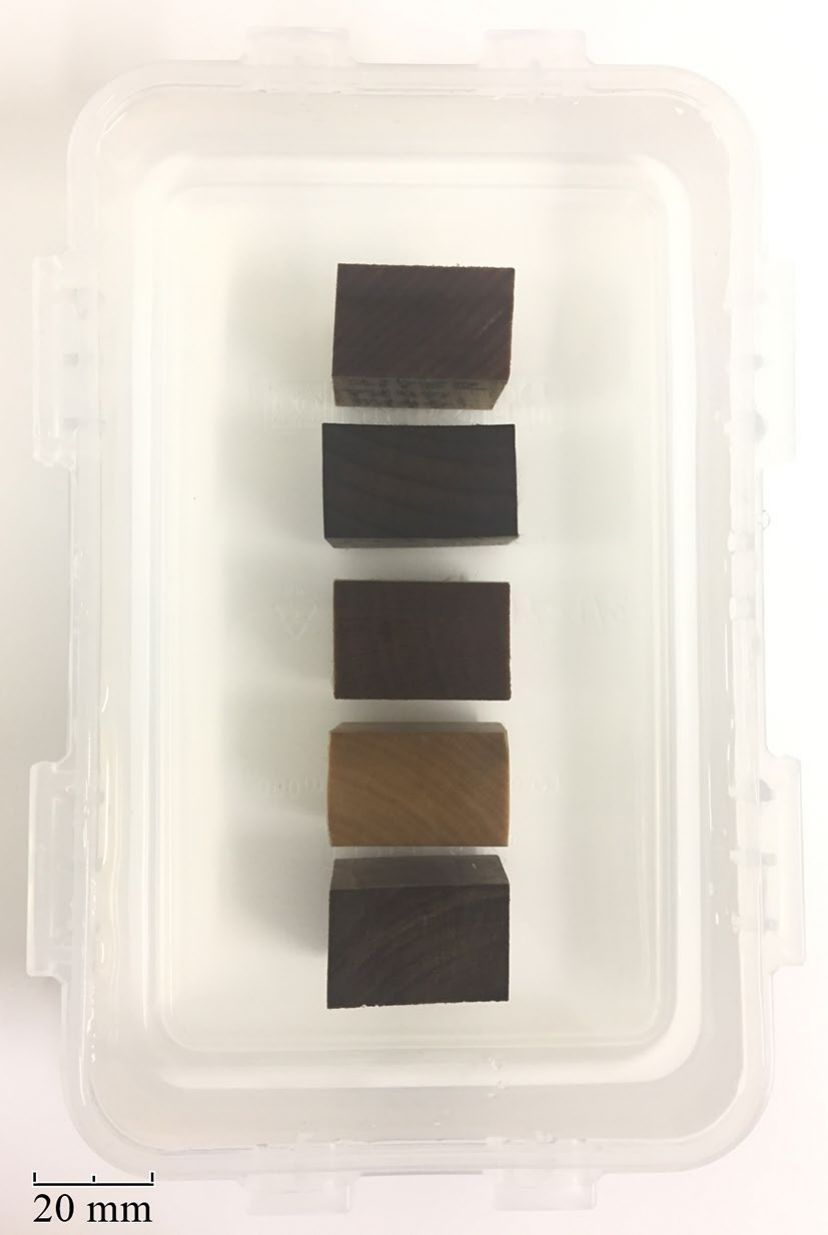
The photograph shows the FA-diffusivity value phantom. The pieces of wood were placed in a container of distilled water that was pressed and fixed with a lid. Five kinds of wood, 20 mm × 30 mm × 30 mm in size, were boiled in distilled water, and the distilled water penetrated into the interior. The wood types are *Gleditsia triacanthos*, *Cinnamomum sieboldii*, *Euodia meliifolia*, *Ilex pedunculosa*, and *Acer palmatum* (from top to bottom)

After harvesting, the wood was formed and dried, followed by storage in a xylarium under controlled room temperature and humidity for several to tens of years. Dry pieces of the ten different wood species, 20 mm × 30 mm × 30 mm in size, were boiled for 30 min in distilled water and stored in a distilled water-filled container for 1 month. As a result of boiling, the air inside the wood expanded and was then discharged. As the temperature decreased after boiling, the wood began to absorb the distilled water into the space previously occupied by the discharged air. In this manner, the air in the wood vessels and fibers was replaced by distilled water. After the 1-month period, any pieces of wood that had been penetrated by the distilled water were fixed in a polypropylene case filled with distilled water (Fig. 1).

#### Fiber phantoms

Tsunooga was used as the fiber phantom material. This fiber is classified as a high-strength polyethylene fiber similar to Dyneema, but with a cross section that is close to a circle, whereas the cross section of Dyneema is oval. The representative cross-sectional diameter of Tsunooga is 12 µm, as described by the manufacturer.

**Fig. 2:**
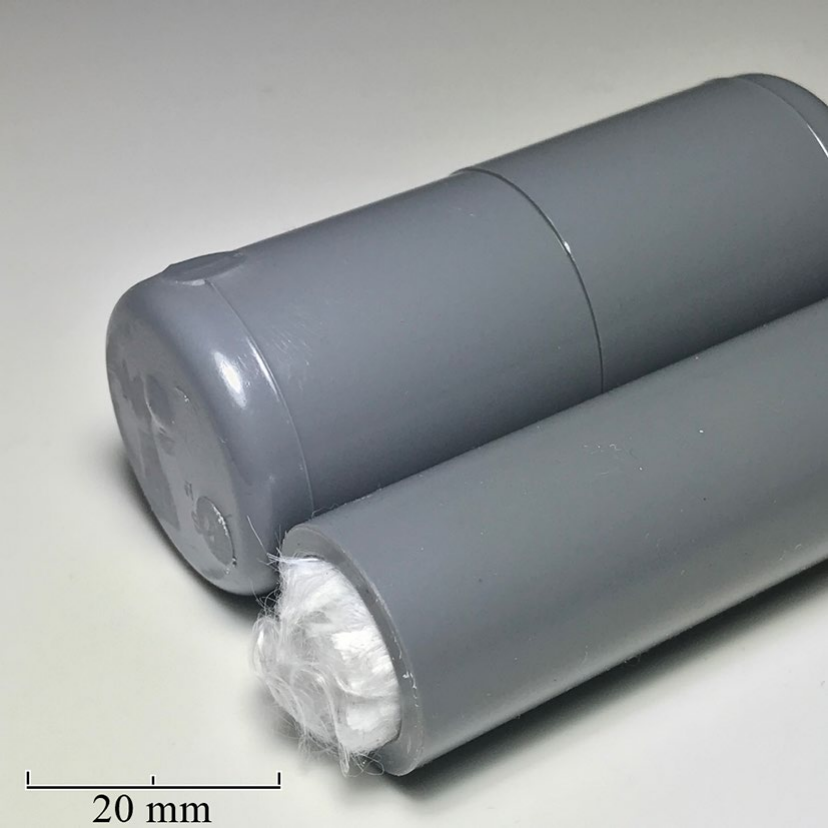
Air was removed from Tsunooga by immersion in water, and the fibers were passed through a polyvinyl chloride pipe that was then sealed with a lid. The cross-sectional diameter of the fibers is 12 µm, and the inside diameter of the pipe is 16 mm

Tsunooga was squeezed by hand in distilled water, and the air bubbles adhering to the fibers were removed. The fibers were passed through a 16-mm-diameter polyvinyl chloride pipe to fix them as a bundle, and then sealed with a lid (Fig. 2). All operations were carried out underwater. Three types of fiber phantoms with different fiber densities were created, with totals of 600,000, 800,000, and 1,000,000 fibers. The number of fibers indicates the nominal number of fibers provided by the manufacturer. The fiber densities were approximately 3,000, 4000, and 5000 fibers/mm^2^, respectively. The fiber density was calculated from the number of fibers in the pipe, and the fiber spacing was not controlled. The differences in fiber density mean that the fiber spacing and anisotropy are also different [17]. Three polyvinyl chloride pipes were placed in a polypropylene case filled with distilled water.

### Imaging

The phantoms were imaged at three facilities. For MR scanners, we used a 3.0-T MAGNETOM Skyra (Siemens Healthineers, Erlangen, Germany) and a 3.0-T MAGNETOM Verio (Siemens Healthineers, Erlangen, Germany), as well as 32-channel head coils. Three MR scanners were used in total for the two systems: Skyra was used at one facility, and Verio was used at two facilities (Verio 1 and Verio 2).

DKI image acquisition was performed using a single-shot spin-echo echo-planar imaging sequence, and other imaging parameters were: echo time, 95.0 ms; repetition time, 7000.0 ms; duration of the motion probing gradients, Skyra 30.2 ms, Verios 1–2 18.1 ms; time between the onset of the motion probing gradients lobes, Skyra 44.1 ms, Verios 1–2 57.6 ms; diffusion directions, 30 directions; b-value, 0–1000–2000 s/mm^2^; bandwidth, 1862 Hz/Px; slice thickness, 2.0 mm; slice gap, 1.0 mm; averages, 2; flip angle, 90 degree; matrix, 122 × 122; voxel size, 2.0 × 2.0 × 2.0 mm^3^; field of view, 250 × 172 mm^2^; acquisition time, 14 min 49 s. Imaging was performed at room temperature at 22 ± 4°C. The direction of the imaging cross section was perpendicular to the wood tissue and Tsunooga fiber direction. Phantoms were installed within the coil such that the wood fibers were perpendicular to the magnetic field and the Tsunooga fibers were parallel to the magnetic field.

#### Phantom imaging using the three MR scanners

For measurement of FA, RK, AK, radial diffusivity (RD), and axial diffusivity (AD) values, imaging was conducted using the FA-diffusivity value phantom, RK–AK value phantom, and fiber phantom. The MR scanners used were Skyra and two Verios equipped with a 32-channel head coil. Phantoms were installed within the coil such that the wood fibers were perpendicular to the magnetic field and the Tsunooga fibers were parallel to the magnetic field.

#### Wood phantom imaging for durability

Phantoms require durability, avoiding changes in properties over a long period. Wood as a natural object has the potential to have its structure changed by wood-decaying fungus. Thus, in the current study, imaging was performed for 9 months to assess the durability of the materials.

The FA-diffusivity and RK–AK value phantoms were each imaged four times at intervals of approximately 3 months. Tsunooga, which is an artificial material, was considered to have a lower possibility of changes in its structure; therefore, it was excluded from the verification of durability in the current study. The MR scanner used was Skyra equipped with a 32-channel head coil. Phantoms were installed within the coil such that the wood fibers were perpendicular to the magnetic field.

#### Influence of phantom orientation in the magnetic field

We examined whether the orientation of the wood fiber and Tsunooga fiber within the magnetic field affected the diffusion metrics. For measurement of FA, RK, and RD values, the FA-diffusivity value phantom, RK–AK value phantom, and fiber phantom were imaged. The MR scanner used was Skyra equipped with a 32-channel head coil. The fiber of each phantom was imaged on the orientation of fibers with respect to the main magnetic field (parallel and perpendicular to the magnetic field).

### Analysis

The FA values were measured from an FA map created [5] using the imaging workstation Syngo.via (VE 11, Siemens Healthineers, Erlangen, Germany). The RK, AK, RD, and AD maps were created with Diffusional Kurtosis Estimator software (Ver. 2.0.6, Medical University of South Carolina, USA) [18], and the RK, AK, RD, and AD values were measured. For all measurements, using ImageJ (Ver. 1.5.2a, National Institutes of Health, Bethesda, USA) and 75% of the phantom area’s region of interest, the average FA, RK, AK, RD, and AD values of the three slices were analyzed.

### Optical microscopic observation

*Gleditsia triacanthos* and *Acer palmatum* were observed using an optical microscope ECLIPSE Ni-U (Nikon, Japan). The specimens for microscopy were prepared by slicing the wood after water absorption at a thickness of 15 µm with an optical microtome RX-860 (YAMATO, Japan) and subsequently immersing them in glycerin. Using the microscope images, the mean major axis and mean minor axis of vessels and wood fibers were measured using ImageJ. The direction of the observation cross section was perpendicular to the wood fiber axis.

## Results

### Phantom imaging using the three MR scanners

The results of the FA, RK, AK, RD, and AD values imaged using the three MRI scanners are shown in the Tables 1, 2, 3, 4, 5, 6. FA-diffusivity value, RK–AK value, and fiber phantoms were analyzed. The data are the mean ± standard deviation (across voxels in a region of interest).

**Table 1:** FA-diffusivity value phantoms imaged using the three MR scanners

Wood species	Fractional anisotropy		
	Verio 1	Verio 2	Skyra
*Gleditsia triacanthos*	0.37 ± 0.06	0.41 ± 0.06	0.38 ± 0.06
*Cinnamomum sieboldii*	0.43 ± 0.06	0.44 ± 0.05	0.50 ± 0.07
*Euodia meliifolia*	0.51 ± 0.05	0.54 ± 0.05	0.59 ± 0.07
*Ilex pedunculosa*	0.64 ± 0.06	0.72 ± 0.06	0.72 ± 0.10
*Acer palmatum*	0.74 ± 0.12	0.78 ± 0.13	0.79 ± 0.12

**Table 2:** Fiber phantoms imaged using the three MR scanners

Fibers	Fractional anisotropy		
	Verio 1	Verio 2	Skyra
600,000	0.46 ± 0.01	0.40 ± 0.03	0.41 ± 0.03
800,000	0.56 ± 0.02	0.55 ± 0.04	0.52 ± 0.03
1,000,000	0.70 ± 0.03	0.68 ± 0.04	0.69 ± 0.02

**Table 3:** Diffusivity of FA-diffusivity value phantoms imaged using the three MR scanners

Wood species	Radial diffusivity (× 10^−3^ mm^2^/s)			Axial diffusivity (× 10^−3^ mm^2^/s)		
	Verio 1	Verio 2	Skyra	Verio 1	Verio 2	Skyra
*Gleditsia triacanthos*	1.36 ± 0.15	1.28 ± 0.23	1.17 ± 0.16	2.50 ± 0.24	2.29 ± 0.16	2.30 ± 0.22
*Cinnamomum sieboldii*	1.21 ± 0.14	1.18 ± ± 0.20	1.07 ± ± 0.20	2.32 ± 0.21	2.13 ± 0.28	2.19 ± 0.20
*Euodia meliifolia*	0.84 ± 0.10	0.89 ± 0.14	0.84 ± 0.15	2.33 ± 0.22	2.26 ± 0.25	2.25 ± 0.14
*Ilex pedunculosa*	0.79 ± 0.17	0.77 ± 0.17	0.78 ± 0.17	2.31 ± 0.23	2.14 ± 0.28	2.12 ± 0.22
*Acer palmatum*	0.96 ± 0.11	0.94 ± 0.21	0.86 ± 0.19	2.08 ± 0.21	1.87 ± 0.26	1.80 ± 0.19

**Table 4:** Diffusivity of fiber phantoms imaged using the three MR scanners

Fiber number	Radial diffusivity (× 10^−3^ mm^2^/s)			Axial diffusivity (× 10^−3^ mm^2^/s)		
	Verio 1	Verio 2	Skyra	Verio 1	Verio 2	Skyra
600,000	1.64 ± 0.11	1.55 ± 0.11	1.54 ± 0.08	2.53 ± 0.12	2.35 ± 0.10	2.28 ± 0.06
800,000	1.35 ± 0.09	1.23 ± 0.08	1.21 ± 0.07	2.52 ± 0.14	2.34 ± 0.08	2.29 ± 0.11
1,000,000	0.83 ± 0.08	0.84 ± 0.10	0.80 ± 0.07	2.65 ± 0.17	2.36 ± 0.10	2.28 ± 0.08

**Table 5:** RK–AK value phantoms imaged using the three MR scanners

Wood species	Radial kurtosis			Axial kurtosis		
	Verio 1	Verio 2	Skyra	Verio 1	Verio 2	Skyra
*Trachycarpus*	0.27 ± 0.06	0.43 ± 0.07	0.26 ± 0.06	0.43 ± 0.06	0.53 ± 0.09	0.38 ± 0.08
*Betula platyphylla*	0.60 ± 0.13	0.78 ± 0.10	0.71 ± 0.10	0.48 ± 0.07	0.59 ± 0.10	0.44 ± 0.09
*Fraxinus longicuspis*	0.93 ± 0.15	1.11 ± 0.18	0.96 ± 0.25	0.72 ± 0.08	0.81 ± 0.15	0.73 ± 0.14
*Eucalyptus*	1.17 ± 0.22	1.39 ± 0.20	1.33 ± 0.22	0.71 ± 0.12	0.81 ± 0.14	0.69 ± 0.12
*Acer mono*	1.59 ± 0.33	1.77 ± 0.35	1.74 ± 0.32	0.78 ± 0.11	0.88 ± 0.16	0.77 ± 0.14

**Table 6:** Fiber phantoms imaged using the three MR scanners

Fibers	Radial kurtosis			Axial kurtosis		
	Verio 1	Verio 2	Skyra	Verio 1	Verio 2	Skyra
600,000	0.54 ± 0.07	0.57 ± 0.08	0.54 ± 0.06	0.20 ± 0.07	0.18 ± 0.06	0.15 ± 0.06
800,000	0.70 ± 0.09	0.79 ± 0.08	0.72 ± 0.10	0.27 ± 0.06	0.21 ± 0.04	0.24 ± 0.08
1,000,000	1.00 ± 0.10	0.99 ± 0.09	1.04 ± 0.11	0.36 ± 0.06	0.29 ± 0.07	0.25 ± 0.06

**Fig. 3:**
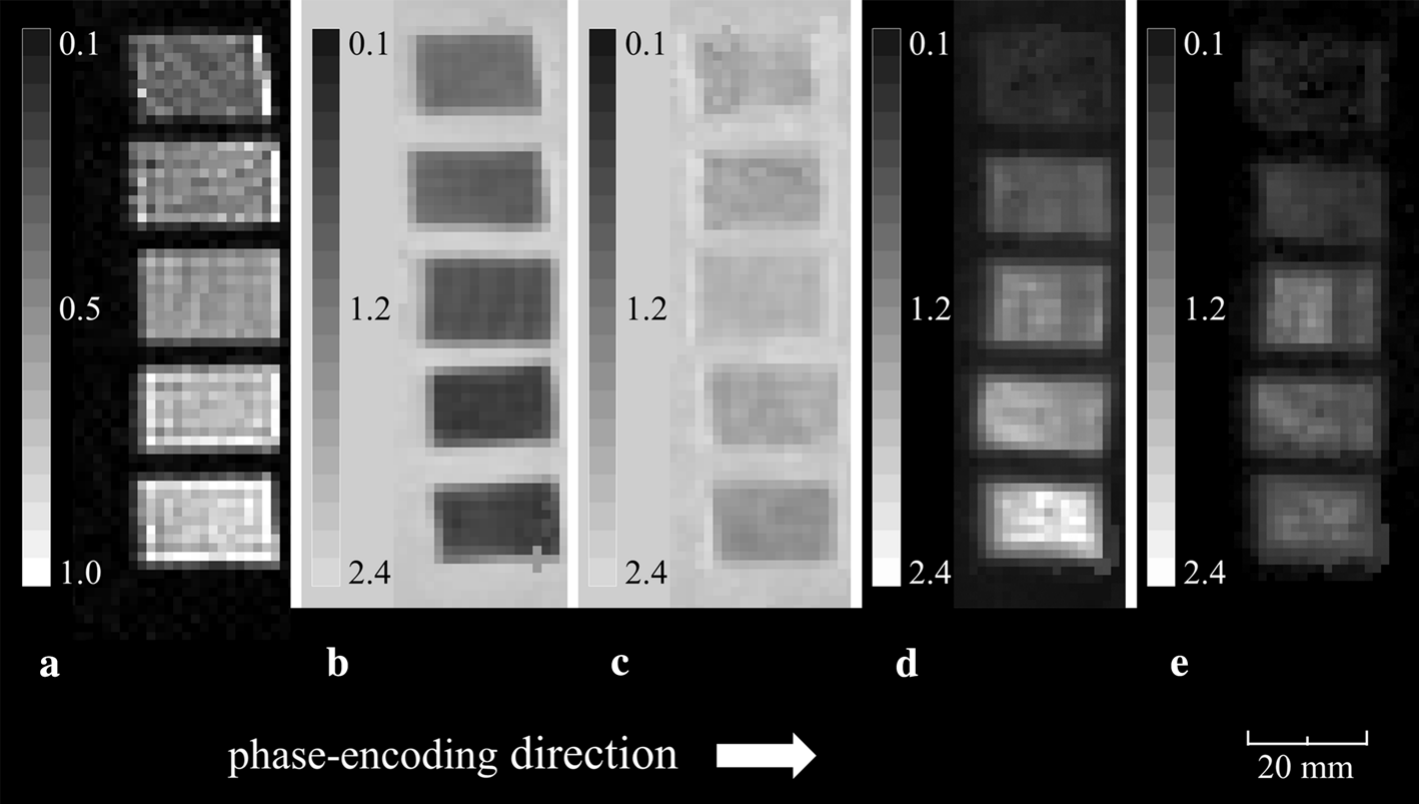
The image shows the FA-diffusivity and RK–AK value phantoms orthogonal to the wood fiber direction. **a** FA map yielded from FA-diffusivity value phantoms. **b** Radial diffusivity map yielded from FA-diffusivity value phantoms. **c** Axial diffusivity map yielded from FA-diffusivity value phantoms. **d** Radial kurtosis map yielded from RK–AK value phantoms. **e** Axial kurtosis map yielded from RK–AK value phantoms

**Fig. 4:**
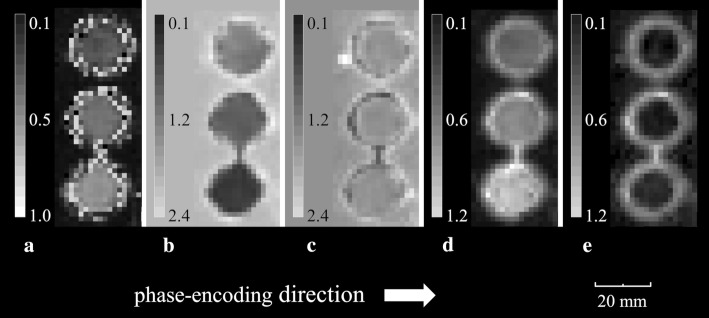
The image shows the fiber phantom orthogonal to the fiber direction. The fiber densities increase from the top to bottom. **a** FA map yielded from fiber phantom. **b** Radial diffusivity map yielded from fiber phantom. **c** Axial diffusivity map yielded from fiber phantom. **d** Radial kurtosis map yielded from fiber phantom. **e** Axial kurtosis map yielded from fiber phantom

The FA-diffusivity value, RK–AK value, and fiber phantom images are shown in Figs. 3, 4.

### Wood phantoms for durability

The results of imaging performed four times at intervals of approximately 3 months are summarized in the Tables 7, 8. The data are the mean ± standard deviation (across voxels in a region of interest). The results of 4 imaging sessions performed in 3-month intervals are expressed as 1st through 4th.

**Table 7:** FA-diffusivity value phantoms imaged for durability

Wood species	Fractional anisotropy			
	1st	2nd	3rd	4th
*Gleditsia triacanthos*	0.37 ± 0.05	0.38 ± 0.06	0.37 ± 0.04	0.37 ± 0.05
*Cinnamomum sieboldii*	0.49 ± 0.05	0.50 ± 0.07	0.51 ± 0.07	0.52 ± 0.08
*Euodia meliifolia*	0.58 ± 0.08	0.59 ± 0.07	0.58 ± 0.09	0.58 ± 0.06
*Ilex pedunculosa*	0.70 ± 0.07	0.72 ± 0.10	0.71 ± 0.09	0.70 ± 0.09
*Acer palmatum*	0.78 ± 0.10	0.79 ± 0.12	0.79 ± 0.11	0.79 ± 0.09

**Table 8:** RK–AK value phantoms imaged for durability

Wood species	Radial kurtosis			
	1st	2nd	3rd	4th
*Trachycarpus*	0.27 ± 0.07	0.26 ± 0.06	0.27 ± 0.06	0.27 ± 0.06
*Betula platyphylla*	0.67 ± 0.10	0.71 ± 0.10	0.67 ± 0.10	0.69 ± 0.10
*Fraxinus longicuspis*	1.01 ± 0.33	0.96 ± 0.25	1.03 ± 0.28	1.07 ± 0.32
*Eucalyptus*	1.31 ± 0.24	1.33 ± 0.22	1.33 ± 0.24	1.31 ± 0.30
*Acer mono*	1.78 ± 0.30	1.74 ± 0.32	1.74 ± 0.34	1.73 ± 0.38

### Influence of phantom orientation in the magnetic field

The Tables 9, 10, 11 show the results of the FA, RK, and RD values imaged in the perpendicular and parallel orientations. The data are the mean ± standard deviation (across voxels in a region of interest).

**Table 9:** FA-diffusivity value phantoms imaged in different orientations

Wood species	Fractional anisotropy		Radial diffusivity (× 10^−3^ mm^2^/s)	
	Perpendicular	Parallel	Perpendicular	Parallel
*Gleditsia triacanthos*	0.38 ± 0.06	0.33 ± 0.03	1.17 ± 0.16	1.46 ± 0.07
*Cinnamomum sieboldii*	0.50 ± 0.07	0.45 ± 0.06	1.07 ± 0.20	1.45 ± 0.12
*Euodia meliifolia*	0.59 ± 0.07	0.51 ± 0.03	0.84 ± 0.15	1.03 ± 0.20
*Ilex pedunculosa*	0.72 ± 0.10	0.61 ± 0.06	0.78 ± 0.17	0.88 ± 0.13
*Acer palmatum*	0.79 ± 0.12	0.76 ± 0.06	0.86 ± 0.19	0.89 ± 0.22

**Table 10:** RK–AK value phantoms imaged in different orientations

Wood species	Radial kurtosis	
	Perpendicular	Parallel
*Trachycarpus*	0.26 ± 0.06	0.26 ± 0.06
*Betula platyphylla*	0.71 ± 0.10	0.64 ± 0.10
*Fraxinus longicuspis*	0.96 ± 0.25	0.93 ± 0.27
*Eucalyptus*	1.33 ± 0.22	1.23 ± 0.23
*Acer mono*	1.74 ± 0.32	1.56 ± 0.26

**Table 11:** Fiber phantoms imaged in different orientations

Fiber number	Fractional anisotropy		Radial diffusivity (× 10^−3^ mm^2^/s)		Radial kurtosis	
	Perpendicular	Parallel	Perpendicular	Parallel	Perpendicular	Parallel
600,000	0.43 ± 0.04	0.41 ± 0.03	1.39 ± 0.09	1.54 ± 0.08	0.64 ± 0.08	0.54 ± 0.06
800,000	0.54 ± 0.03	0.52 ± 0.03	1.16 ± 0.14	1.21 ± 0.07	0.82 ± 0.11	0.72 ± 0.10
1,000,000	0.73 ± 0.04	0.69 ± 0.02	0.68 ± 0.17	0.80 ± 0.07	1.22 ± 0.15	1.04 ± 0.11

### Optical microscopic observation

The measurement results of the diameters of vessels and wood fibers are shown in Table 12.

**Table 12:** Microscopic analysis results of *Gleditsia triacanthos* and *Acer palmatum*

Wood species	Vessels (µm)		Wood fibers (µm)	
	Mean major axis	Mean minor axis	Mean major axis	Mean minor axis
*Gleditsia triacanthos* (FA 0.38 ± 0.06)	205 ± 47	147 ± 33	8 ± 3	5 ± 2
*Acer palmatum* (FA 0.79 ± 0.12)	59 ± 10	42 ± 8	12 ± 3	8 ± 3

**Fig. 5:**
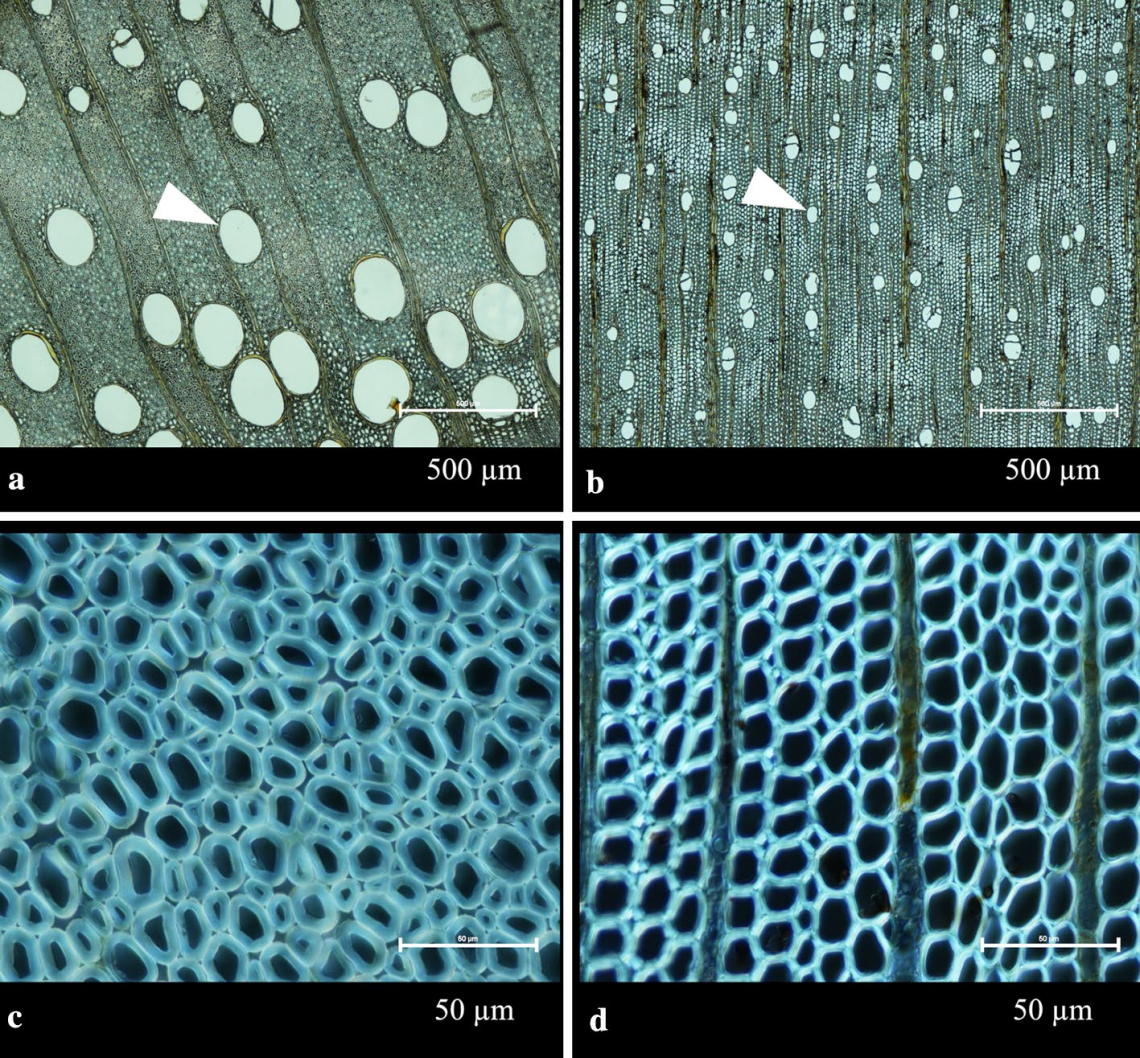
Optical microscope images show vessels and wood fibers, in a slice which is orthogonal to the wood fiber direction. The respective FA values of *Gleditsia triacanthos* and *Acer palmatum* were 0.38 and 0.79. The FA values are cited from Table 7 of the second imaging session. Vessels and wood fibers were probably filled with glycerin. Transmitted light was used for **a** and **b**, whereas differential interference contrast was used for **c** and **d**, resulting in a dark appearance inside the vessels and wood fibers due to the differential interference filter. **a** Vessels of *Gleditsia triacanthos* was observed (arrow head). **b***Acer palmatum* was confirmed to have smaller diameter vessels than *Gleditsia triacanthos* (arrow head). **c** The magnified image of *Gleditsia triacanthos* displays wood fibers. **d** The magnified image of *Acer palmatum* displays wood fibers

The results of optical microscopic observations are shown in Fig. 5.

## Discussion

Different results were obtained with the three MR scanners (Tables 1, 2, 3, 4, 5, 6). Regarding the conditions of measurement, there were differences in the magnetic field gradients between Skyra and Verio, in the durations of the motion probing gradients, and in the time between the onset of the motion probing gradient with Skyra and Verio. Other factors having the potential to lead to different results of measurement are measurement error and changes in the phantom materials. It was previously reported that changes in diffusion time can affect the displacement distribution profiles of water [19]. In the present study, the gradient pulse duration was identical for the two Verios, but the diffusion metrics yielded between these two MR scanners were different. Between Skyra and Verio, the gradient pulse duration differed and the diffusion metrics obtained also differed. Thus, there was no definite association of diffusion metrics with the gradient pulse duration. Further study is needed to identify the reason why different results were yielded by the 3 MR scanners in the present study.

Performing the imaging four times over a period of 9 months did not affect the differences in FA and RK values (Tables 7, 8). Therefore, any structural changes in the wood over the 9-month period were determined to be minimal. However, further investigation to confirm these results is necessary.

Changing the phantom orientation within the magnetic field resulted in changes in diffusion metrics. (Tables 9, 10, 11) FA and RK were always higher when the fiber orientation within magnetic field was perpendicular, unlike when the orientation was parallel. Follow-up studies are needed to identify the cause for the observed findings. At present, we can only speculate: These changes may be due to cross terms between diffusion and background gradients caused by susceptibility differences. Signal attenuation due to a similar cross term has been reported previously [20]. Another speculation: It is possible that the diffusion gradient direction and the phantom orientation differed between Verio and Skyra. The increase in the number of directions could have altered the diffusion metrics.

The diffusion gradient pulses can alter the magnetic flux density, possibly leading to formation of eddy currents. Interleaved echo-planar techniques were used to calibrate and compensate for these geometric distortions. The eddy current induced artifact was, thus, reduced [21]. Eddy currents are often observed as high FA values at the border of the object; therefore, the high FA values at the wood interface, as shown in Fig. 3a, may be attributable to eddy currents.

The data from this analysis were compared with those from previous reports on values of the human brain: FA; 0.14 (caudate head)–0.83 (splenium of corpus callosum), RD; 0.31 (splenium of corpus callosum)–1.57 (caudate head), RK; 0.59 (caudate head)–2.72 (splenium of corpus callosum) [7, 22]. In this study, the maximum and minimum of diffusion metrics were determined with the wood phantom. Each of these values represents the average of the values across the voxels in a region of interest of three slices. The values determined with the fiber phantom were also obtained from an analysis conducted in a way similar to the values from the wood phantom. The values were: wood: FA; 0.38–0.79, RD; 0.86–1.17, RK; 0.26–1.74, and fiber: FA; 0.41–0.69, RD; 0.80–1.54, RK; 0.54–1.04. Diffusion parameters in human brain tissue cover a considerable value range; each type of wood and different fiber densities also has different diffusion metrics.

Due to financial and technical limitations, it is difficult to produce phantoms that are large and flexible using glass capillaries and fibers. Although they can be used with compact MRI scanners, there are phantoms that are too small to be used with clinical MRI scanners [23]. As a thin piece of wood can bend to some extent, it can be made into phantoms with curved parts. Additionally, large wood pieces can be used to create large phantoms.

The vessels of wood serve as the route for water flow and are likely to be filled with water after boiling. Water may also penetrate wood fibers. The vessel diameter of *Gleditsia triacanthos* was larger than that of *Acer palmatum*, thus its FA value was smaller than that of *Acer palmatum*. The smaller FA value of *Gleditsia triacanthos* despite the smaller wood fiber diameter of *Gleditsia triacanthos* as compared with *Acer palmatum* may be explained by the greater contribution of vessels than wood fibers to anisotropy.

Ten kinds of wood were readily available and can be used as phantom materials. The main objective of the present study was not to identify the optimum wood species for phantom material, but to clarify whether the wood is suitable as an anisotropic phantom material. Each of the 10 types of wood had different diffusion metrics. Although phantoms were divided into FA-diffusivity value phantoms and RK–AK value phantoms in the present study, whichever type of wood is available may be used without consideration of this distinction. There are many wood species available, and they differ according to country and region. Therefore, wood phantom users in other countries should not necessarily use the specific wood species used in the present study; rather, they should use wood species that are available to them in their country.

Dyneema has been frequently used as a fiber phantom material, which has made it possible to create fibers crossing each other [24, 25]. Previous studies have reported that differences in fiber density are associated with changes in FA values [26]. Similar to Dyneema, Tsunooga is composed of polyethylene fibers, which are known to be hydrophobic. We, therefore, consider the contribution of water molecules within the fibers to diffusive motion to be small. Protons move between each fiber, and the fibers hinder diffusion. The fibers were difficult to bunch together by hand in the current study; but some studies have proposed machine-made fiber phantoms to combat this issue [27].

Our study had several limitations. First, the long-term durability was not sufficiently examined. Technologies, such as wood sterilization and sealing with resin, may improve their durability. Secondly, the susceptibility for artifacts was not assessed; therefore, artifacts may have influenced the results. Finally, wood availability and prices were not investigated because they differ from country to country. Using wood that is easy to obtain at affordable prices may be an effective use of said wood.

In conclusion, five kinds of wood had different FA, RK, AK, RD, and AD values between three MR scanners. The wood durability for a period of 9 months was also confirmed. Processing was possible without specialized equipment. Wood is promising as an anisotropic phantom material for restricted diffusion.

Tsunooga had different FA, RK, AK, RD, and AD values depending on fiber densities. This fiber is promising as an anisotropic phantom material for hindered diffusion as an alternative to Dyneema.
